# DFT-Based Analysis on Structural, Electronic and Mechanical Properties of NiCoCr Medium-Entropy Alloy with C/N/O

**DOI:** 10.3390/ma18194494

**Published:** 2025-09-26

**Authors:** Shuqin Cheng, Yunfeng Luo, Yufan Yao, Yiren Wang, Fuhua Cao

**Affiliations:** 1 Key Laboratory for Nonferrous Materials (MOE), School of Materials Science and Engineering, Central South University, Changsha 410083, China; 233112103@csu.edu.cn (S.C.); 243112145@csu.edu.cn (Y.L.); 8204222119@csu.edu.cn (Y.Y.); 2 National Key Laboratory for Powder Metallurgy, Central South University, Changsha 410083, China

**Keywords:** NiCoCr alloy, solution energies, stacking fault energy, first-principles study

## Abstract

This study employs first-principles calculations combined with the Special Quasirandom Structure (SQS) technique to investigate the impact of three interstitial elements C, N, and O, on the mechanical properties and stacking fault energy (SFE) of NiCoCr medium-entropy alloys. The results indicate that non-metallic O, C, and N tend to occupy octahedral interstitial sites, which can effectively release stress concentration and enhance the strength and deformability of the material. Differential charge density analysis shows that the dissolution of C, N, and O significantly alters the surrounding electronic environment, strengthening the interaction between solute atoms and metal atoms, thereby hindering dislocation glide and increasing the strength and hardness of the material. Elastic property analysis indicates that NiCoCr alloys doped with C, N, and O exhibit good ductility and anisotropic characteristics. Furthermore, the study of stacking fault energy reveals that the doping with C, N, and O can significantly increase the stacking fault energy of NiCoCr alloys, thereby optimizing their mechanical properties. These findings provide theoretical evidence for the design of advanced high-entropy alloys that combine high strength with good ductility.

## 1. Introduction

Multi-principal element alloys (MPEAs), including high-entropy alloys (HEAs) and medium-entropy alloys (MEAs), typically exhibit superior mechanical properties [1]. MPEAs with fcc structures are believed to possess better ductility and low-temperature fracture toughness than those comprising bcc structures. Among which, NiCoCr have garnered significant attention due to their excellent mechanical properties, including high tensile strength (~1.0 GPa), exceptional elongation (~50%), and excellent fracture toughness (~200 MPa·m^1/2^ [2,3,4,5,6]). However, single-phase-fcc NiCoCr alloys exhibit relatively low stacking fault energy (SFE) and their inferior yield strength has limited their application in actual structural components. More critically, the multifunctional attributes of this alloy system—spanning corrosion resistance, catalytic activity, and tunable electronic structure—remain largely unexplored, thereby constraining its potential value in functional material applications. To tackle this issue, traditional strengthening strategies, including grain boundary strengthening [7,8,9], dislocation strengthening [10,11], and precipitation strengthening [12,13,14]^,^ have been applied to NiCoCr alloys. However, these methods often struggle to balance strength and toughness simultaneously and result in a significant reduction in ductility.

Several studies have shown that the non-metallic interstitial atoms (such as C, N, O) in a NiCoCr matrix can cost-effectively optimize the mechanical properties. The inherent severe lattice distortion in MEAs may provide better accommodation for interstitial atoms and reduces the formation of brittle ceramic phases [15]. For instance, Shang et al. reported that the sole addition of carbon (<0.75 at%) in a NiCoCr alloy matrix led to a yield strength improvement without the formation of any carbides while maintaining the ductility rate at as high as about 75% [16]. Anh et al. obtained yield strengths of 747.5 MPa and 832.2 MPa for 0.25 at% and 0.75 at% carbon-doped NiCoCr alloys, respectively, through additive manufacturing [17]. They further enhanced the yield strength to 872.7 MPa for 0.75 at% C-doped alloys by post heat treatment [18]. The enhancement was attributed to solid solution-strengthening and precipitation-strengthening effects of in situ Cr-rich M23C6 carbides while the formation of undesired carbides led to a loss in ductility. Interstitial N-doping is expected to be favored over C-doping due to its usually higher solubility and slower kinetics in the formation of nitrides compared to the formation of carbides. Moravcik et al. incorporated 0.5 at% N into NiCoCr alloys using spark plasma sintering (SPS), increasing the yield strength by 24–33% without degrading the elongation [19]. Dong et al. employed plasma arc melting technology to fabricate N-doped NiCoCr alloys without forming nitride inclusions. The improved yield strength originated from the localized short-range ordered N atoms, which can effectively facilitate the dislocation storage and increase the lattice friction through first-principles calculations [20]. Nitrogen doping increases the stacking fault energy, suppresses twinning, and promotes the multiplication of dislocations, contributing to an enhanced strength–ductility balance. Recently, researchers have begun to focus on the potential impact of oxygen in NiCoCr-based MEAs as well. Song et al. [21] reported that doubly enhancing the yield strength of (NiCoCr)_94_O_6_ compared to O-NiCoCr alloy resulted in the segregation of oxygen atoms which then caused dislocation–twin interaction. Further DFT calculations suggested a relatively low diffusion barrier for oxygen interstitials in the NiCoCr matrix, which improves its ability to accommodate and release severe stress concentration caused by local lattice distortion [22]. Although experiments and theories on doping-NiCoCr MEAs have advanced, there is still a lack of understanding of the underlying strengthening mechanism behind the interstitial alloying and their interstitial effects. Furthermore, the incorporation of interstitial elements can markedly modulate electronic transport, magnetism, and surface reactivity by altering local charge density, inducing lattice distortion, or reconfiguring bonding characteristics and properties that are pivotal for advances in sensors, catalysis, and energy-related materials [23,24].

Density functional theory (DFT)-based first-principles calculations have been considered as strong tools [25,26,27,28] in revealing the interactions between interstitial atoms and metallic matrix elements at the atomic level [29,30,31]. In this study, we employed the first-principles method to investigate the effect of interstitials C, N, and O in NiCoCr MEAs on defect structures and energetics. The atomic structures, elastic properties, generalized stacking faults, and charge redistribution were systematically studied to provide a comparative theoretical analysis and modeling of the strengthening mechanism and deformation behaviors. The findings can help understand the solid solution-strengthening mechanism of medium-entropy alloys and provide guidance for the development of advanced metallic alloys with a unique combination of strength and ductility.

## 2. Calculation Details

### 2.1. DFT Calculation

All the first-principles density functional theory (DFT) calculations were performed using Vienna ab initio Simulation Package (VASP) [32] with the plane-wave basis sets. The electron–core interaction was described by the BlÖchl projector-augmented wave method (PAW) within the frozen-core approximation as implemented in VASP [33]. The Perdew–Burke–Ernzerhof (PBE) version of generalized gradient approximation was employed for the exchange-correlation functionals [34].

**Figure 1:**
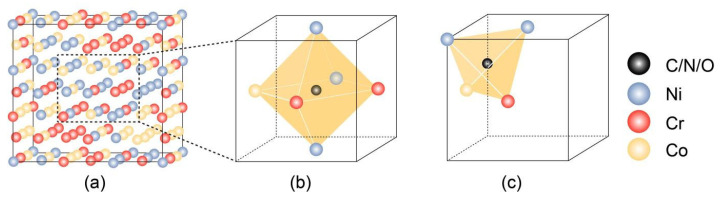
(**a**) NiCoCr SQS model. (**b**) The distribution of carbon, nitrogen, or oxygen interstitials in different octahedral sites in NiCoCr alloy. (**c**) The distribution of carbon, nitrogen, or oxygen interstitials in different tetrahedral sites in NiCoCr alloy.

The present study employs the first-principles method in combination with the Special Quasirandom Structure (SQS) to construct a 3 × 3 × 3 face-centered cubic (fcc) NiCoCr matrix, as seen in Figure 1a. The term “Special Quasirandom Structures” refers to a mathematical model used to generate structures for disordered alloys. These structures exhibit statistical properties that closely mimic those of a truly random alloy, making them particularly useful for computational simulations and theoretical studies. SQSs are designed to approximate the correlation functions of an infinite random alloy more accurately than traditional random structures, even for small system sizes. This approach allows for the application of accurate electronic structure methods to calculate properties of random alloys, such as structural, optical, and thermodynamic properties.

A high-energy cutoff of 460 eV was adopted for all the plane-wave basis sets and a 3 × 3 × 3 Monkhorst–Pack K-mesh for the Brillouin zone integrations, which can sufficiently achieve a reasonable total energy convergence of <1 meV/atom for the 3 × 3 × 3 fcc NiCoCr supercell (108 atoms). During relaxation calculations, the supercells were fully relaxed on both the cell volume and shape. In our calculation, the lattice constant of the NiCoCr matrix is determined to be 3.517 Å, which is in good agreement with the experimental value of 3.560 Å [35].

### 2.2. Formation Energy Calculation

The dissolution process of the concerned interstitial elements C, N, and O in the NiCoCr medium-entropy alloy is evaluated from first-principles energetics. The potential interstitial sites are depicted in Figure 1b,c. We define the formation energy for C, N, and O at interstitial sites in the NiCoCr host as

$$\Delta E_{(C/N/O)}^{S}=E_{NiCoCr+C/N/O}-E_{NiCoCr}-\mu_{X}$$

where $E_{NiCoCr+C/N/O}$ is the total energy of the 3 × 3 × 3 supercell of the fcc NiCoCr supercell embedded with a single isolated solute atom of C, N, or O at an interstitial site.$\;E_{NiCrCo}$ is the total energy of the fcc NiCoCr supercell. $\mu_{X}$ represents the chemical potential of X atoms, where O/N/C is taken as the molecular oxygen, molecular nitrogen, and graphite, respectively.

During the relaxation process, the atoms moved from their initial fixed positions to new positions to minimize the energy of the system. Additionally, the lattice constants were allowed to be optimized during the calculation, which may have led to the significant lattice distortion. Recently, a parameter ∆d [36] based on DFT calculations was proposed to quantitatively describe the degree of local lattice distortion in HEAs, as expressed by

$$∆d=\sqrt{{\left(x_{0}-x_{i}\right)}^{2}+{\left(y_{0}-y_{i}\right)}^{2}+{\left(z_{0}-z_{i}\right)}^{2}}$$

where ($x_{0}$, $y_{0}$, $z_{0}$) and ($x_{i}$ $y_{i}$, $z_{i}$) are the Cartesian coordinate positions of the atoms before and after structural relaxation, respectively.

### 2.3. Stacking Fault Energy Calculation

Initially, a CrCoNi slab with an fcc structure (comprising 360 atoms) was created by sequentially stacking 10 close-packed (111) atomic planes. In the simulation cell, a vacuum layer with a thickness of 1.6 nm was inserted in the direction perpendicular to the slab (z direction) to separate each slab from the adjacent slab in the next periodic unit (to minimize the interaction between slabs). The atomic configuration of the slab was geometrically relaxed in all three directions. Subsequently, an intrinsic stacking fault was generated by shifting the top 4 layers along the Burgers $<11\overset{-}{2}>$ vector of the Shockley $b_{S}=a/6<11\overset{-}{2}>$ partial dislocation. We define the stacking fault energies (SFEs) for C, N, and O at different sites in the NiCoCr host as

$$\gamma_{isf}=(E_{isf}-E_{0})/S_{0}$$

where $E_{isf}$ and $E_{0}$ represent the total energy of the fault and the original supercell, respectively, and $S_{0}$ is the area of the stacking fault.

## 3. Results

### 3.1. Atomic Distribution of Solid Solution

**Figure 2:**
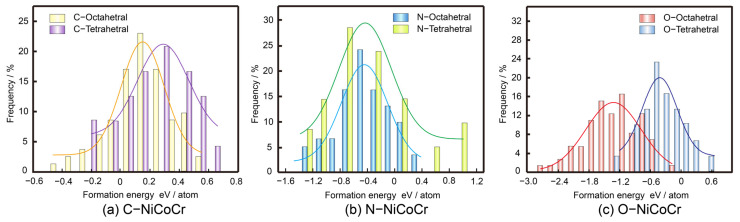
Distribution of formation energy of C, N, and O interstitials at different octahedral and tetrahedral sites in NiCoCr alloy.

**Figure 3:**
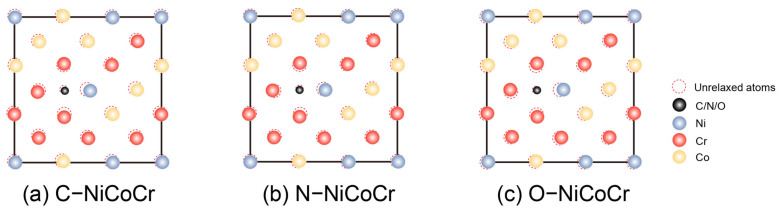
The change in atomic positions before and after relaxation, with the red circles indicating the positions of the atoms after relaxation.

The incorporation of non-metallic atoms is initially investigated by calculating the formation energies at potential interstitial sites. From Figure 2, it can be seen that N and O interstitial elements at either octahedral or tetrahedral interstices are predicted to be exothermic, indicating a high likelihood of solid solution. C, N, and O all prefer to occupy octahedral interstitial sites with larger interstitial volume. The metal atoms Ni, Co, and Cr are randomly distributed after modeling with the SQS method. Obvious lattice distortion can be observed after the solid solution as shown in Figure 3. The degree of lattice distortion (∆d) for C-, N-, O- NiCoCr alloy is calculated to be 0.015, 0.039, and 0.091 Å, respectively. The distribution of non-metallic atoms is found to rely on their neighboring Cr atoms in the matrix from statics, since they tend to stay in chromium-rich lattice area [22].

### 3.2. Elastic Constants

**Figure 4:**
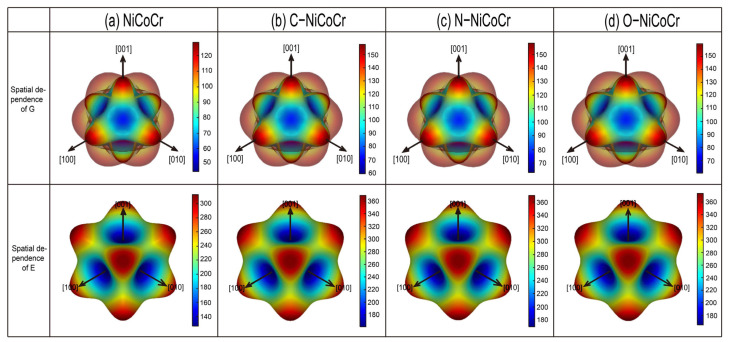
Three-dimensional view of directional dependence of E and G of NiCoCr, C/N/O-NiCoCr MPEAs.

The elastic constants are calculated to describe the mechanical behavior of the solid solution alloys. The numerical results for the elastic constants C_11_, C_12_, C_44_, and Pugh’s ratio obtained from the stress–strain method are shown in Table 1. The NiCoCr matrix is in agreement with the results reported in the existing literature. It is worth noting that after the introduction of C, N, or O atoms, the elastic constants C_11_ and C_44_ both show a significant increase, while C_12_ slightly decreases. Furthermore, the interstitial doping has enhanced the ductility of the NiCoCr alloy, among which C exhibits the most favorable Pugh’s ratio. The mechanical parameters and anisotropic characteristics of the NiCoCr medium-entropy alloy, including three-dimensional directional dependence of E and G for NiCoCr, and C/N/O-NiCoCr, are illustrated in Figure 4. Non-metallic doping can enhance the three-dimensional directional dependence of E and G for the NiCoCr alloy. The computational results indicate that the G and E of the C/N/O-NiCoCr alloys both show a significant increase, while the differences in the impact of various doping elements (C, N, O) on the mechanical properties of the NiCoCr base alloy are relatively small.

### 3.3. Stacking Fault Energy

**Figure 5:**
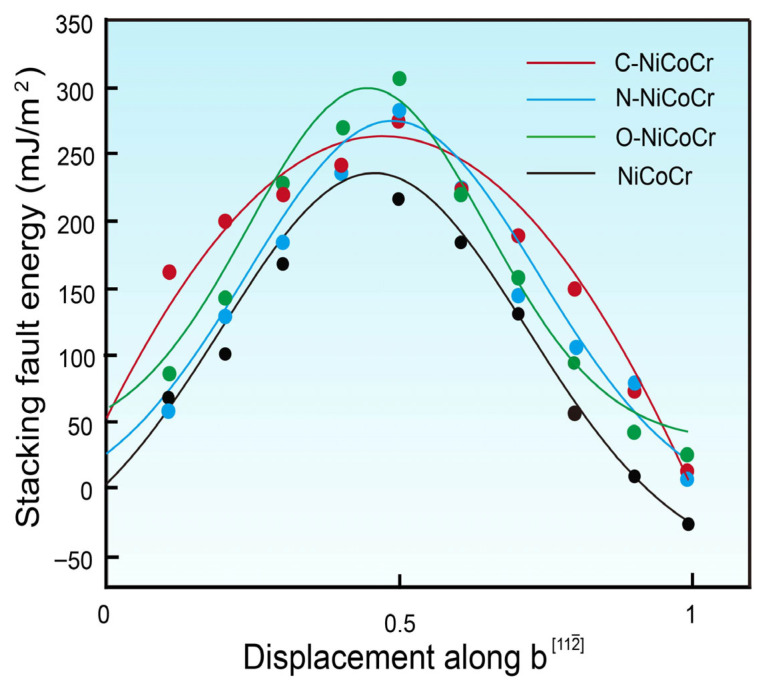
G_SFE_ curves for twinning nucleation in the plane with partial dislocation of the FCC CrCoNi-based MEAs.

Figure 5 shows the stacking fault energy as a function of dislocation displacement (along b) for the NiCoCr, C/N/O-NiCoCr alloys. The detailed values and references are summarized in Table 1. The calculated data for the NiCoCr ternary alloy are in good agreement with the previous theoretical calculation results; however, the calculated results for the NiCoCr(C/N) system formed after the introduction of C/N elements show some deviation from experimental observations, which may be attributed to the difference between the experimental process’s C/N element segregation behavior and the ideal solid solution assumption. Doping with O increases the stacking fault energy of the NiCoCr alloy from −35 mJ/m^2^ to 34 mJ/m^2^, a change of approximately 69 mJ/m^2^. Doping with C increases the stacking fault energy by about 54 mJ/m^2^, while doping with N increases it by about 58 mJ/m^2^. This indicates that doping with O significantly increases the stacking fault energy of the NiCoCr alloy. The unstable stacking fault energy (*γ_us_*) reflects the saddle point energy of the dislocation core decomposition on the generalized stacking fault energy surface. According to our calculations, *γ_us_* increases with the incorporation of interstitial atom and follows C < N < O. The MEA has a simple microstructure without a second phase formation; the addition of 1 at.% C/N/O can induce potential enhancement in strength.

## 4. Discussion

This study has conducted an in-depth analysis of the effects of C, N, and O on the NiCoCr medium-entropy alloy using first-principles calculations. Compared with the doping of C and N atoms, oxygen atoms are more readily soluble in the NiCoCr alloy. Doping with C/N/O does not lead to the formation of new ordered structures, but it does cause lattice distortion, implying that some Ni, Co, and Cr atoms deviate from their original positions. We found that the O interstitial elements in the distorted lattice had relative low formation energies, suggesting that when there is a sufficient amount of O elements in the NiCoCr alloy, O elements will exist simultaneously in octahedral interstices. Due to the size effect, local stress fields will be generated around the lattice. These stress fields will hinder the movement of dislocations, thereby enhancing the strength of the material. Additionally, interstitial impurities cause lattice distortion, increasing the energy of the lattice, and thus affecting the stability of the material. The doping of high concentrations of O interstitial atoms, on one hand, generates a high density of severe local lattice distortions, effectively hindering the movement of dislocations throughout the lattice and accumulating strain energy for strengthening; on the other hand, the migration of oxygen enhances the ability to adapt to and release severe stress concentration, which is rarely reported in FCC materials [22]. On the other hand, doping can alter the stacking fault energy of the alloy, inhibiting the nucleation and growth of deformation twins, thus reasonably explaining the low proportion of deformation twin interfaces in the plastically deformed CoCrNi alloy. Subsequently, an analysis is conducted in conjunction with the differential charge density of the doped C, N, and O atoms.

**Figure 6:**
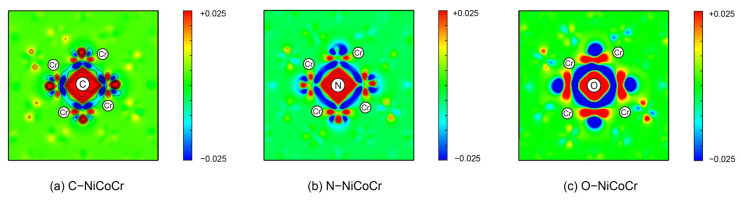
Differential electron densities in the units of electrons/Å^3^ in the (001) plane for an octahedral interstitial C, N, O.

**Figure 7:**
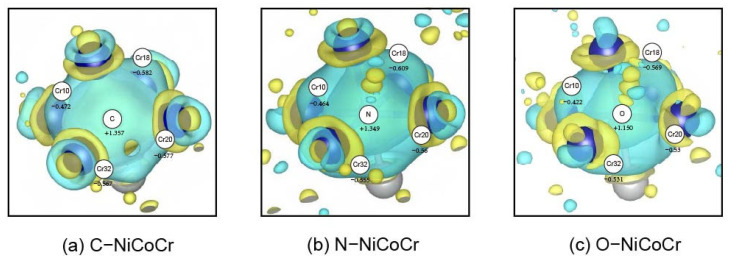
In the (001) plane, the electron transfer situation of octahedral interstitial atoms (C, N, O) was calculated through Bader charge analysis, with the unit being electrons/Å^3^. Here, Cr10 represents the 10th Cr atom in the system.

As can be seen from Figure 6, significant charge accumulation can be found around all three elements with an electron depletion of the metal bonds between the outer metal layers correspondingly. As shown in Figure 7, the Bader charge analysis results indicate that the electrons are mainly concentrated around the interstitial atoms, suggesting that the interstitial atoms attract electrons due to the electronegativity effect. At the same time, the electrons around the Cr atoms are lost and transferred to the interstitial atoms. Among them, the electrons around C accumulate the most significantly, and the charge loss of the metal atoms is the greatest, while the influence of O is relatively weak. This indicates that the interstitial solid solution of the three atoms has gradually transformed the original metallic bonds between the surrounding metals into stronger ionic bonds between the solute atoms and the surrounding metal atoms. Such a transformation can contribute to strengthening the enhancement by increasing the energy required for atoms to separate, thereby hindering dislocation glide [41]. The local charge density of the solid-solutioned C atom significantly increases and shows a certain directionality. N atoms also exhibit strong charge redistributions, but their intensity and directionality are lower than that of C atoms. O atoms have the weakest charge interaction with the surrounding metal atoms compared to C and N elements and hardly show any directionality. The charge redistribution patterns indicate that the electron bonding nature of non-metallic doping follows C > N > O.

The introduction of interstitial atoms C, N, and O can enhance the mechanical properties of the NiCoCr alloy. Computational results indicate that the shear modulus, Young’s modulus, and stacking fault energy all show an upward trend, suggesting that the stiffness and load-bearing capacity of the alloy have been effectively strengthened. The strengthening effect of O atoms on the NiCoCr alloy is the most pronounced, followed by N atoms, with C atoms being relatively weaker. This may be related to the strength of the interaction between the interstitial atoms and the matrix metal atoms [42]. The DCD results indicate that carbon atoms have the strongest electronic interaction with the matrix metal atoms, and thus the strongest impediment to the dislocation glide. On the other hand, the calculation of formation energy shows that oxygen atoms are more readily soluble in the NiCoCr alloy compared to carbon and nitrogen atoms, indicating that at the same concentration of interstitial atoms, the solubility of oxygen atoms is higher, leading to a more significant enhancement of material properties. Future research can further explore the impact of different concentrations of interstitial atoms on the microstructure and mechanical properties of the NiCoCr alloy, as well as optimize the alloy composition design to achieve superior mechanical properties.

## 5. Conclusions

This study employed first-principles calculations combined with the Special Quasi-random Structure (SQS) technique to systematically investigate the effects of interstitial elements C, N, and O on the structural, electronic, and mechanical properties of NiCoCr medium-entropy alloys (MEAs). The key findings are summarized as follows:

(1) Interstitial site preference and lattice distortion: C and N preferentially occupy octahedral interstitial sites, while O exhibits a higher diffusion rate between octahedral and tetrahedral sites, effectively alleviating stress concentration. The dissolution of these interstitial atoms causes significant lattice distortion, with O causing the most noticeable distortion (16.82%), followed by N (10.84%) and C (9.27%).
(2) Mechanical properties: The introduction of interstitial atoms increases the elastic constants and SFE, indicating an improvement in stiffness and resistance to plastic deformation. O doping leads to the most significant increase in SFE (69 mJ/m^2^, from −35 mJ/m^2^ to 34 mJ/m^2^), followed by N (58 mJ/m^2^) and C (54 mJ/m^2^). It optimizes the strength–ductility balance of the alloy by suppressing twinning and promoting dislocation glide.
(3) Electronic interactions: Differential charge density analysis shows that C, N, and O atoms significantly alter the surrounding electronic environment, strengthening the ionic bonds between the solute and metal atoms. C exhibits the strongest directional charge interactions, indicating covalent characteristics, while O shows the weakest interactions.

## Figures and Tables

**Table 1:** Based on first-principles calculations, the results of the elastic constants (C_11_, C_12_, C_44_), Pugh’s ratio, and stacking fault energy for NiCoCr-based alloys. *γ_isf_*—intrinsic stacking fault; *γ_us_*—unstable stacking fault.

	C_11_	C_12_	C_44_	Pugh’s Ratio	*γ_isf,_* mJ/m^2^	*γ_us,_* mJ/m^2^
NiCoCr	245 ± 4	155 ± 6	129 ± 3	2.40	−35	245
	(250 ^calc^ [37])	(175 ^calc^ [37])	(100 ^calc^ [37])		(−24 ^calc^ [3])	(264 ^calc^ [3])
					(−26 ^calc^ [38])	(313 ^calc^ [38])
					(−60 ^calc^ [39])	(269 ^calc^ [39])
C-NiCoCr	264 ± 5	146 ± 7	158 ± 2	2.45	19	264
					(29 ^calc^ [40])	
N-NiCoCr	269 ± 6	144 ± 7	158 ± 4	2.41	23	278
					(42 ^expt^ [40])	
O-NiCoCr	270 ± 7	150 ± 6	159 ± 4	2.40	34	295

## Data Availability

The original contributions presented in this study are included in the article. Further inquiries can be directed to the corresponding authors.
